# Acrylonitrile–Acrylic Acid Copolymer Ultrafiltration Membranes for Selective Asphaltene Removal from Crude Oil

**DOI:** 10.3390/membranes13090775

**Published:** 2023-09-01

**Authors:** Alexey A. Yushkin, Alexey V. Balynin, Alexandra P. Nebesskaya, Elena V. Chernikova, Dmitriy G. Muratov, Mikhail N. Efimov, Galina P. Karpacheva

**Affiliations:** 1 A.V. Topchiev Institute of Petrochemical Synthesis, Russian Academy of Sciences, 29 Leninsky Prospekt, 119991 Moscow, Russia; ab@ips.ac.ru (A.V.B.); nebesskaya@ips.ac.ru (A.P.N.); chernikova_elena@mail.ru (E.V.C.); muratov@ips.ac.ru (D.G.M.); efimov@ips.ac.ru (M.N.E.); gpk@ips.ac.ru (G.P.K.); 2 Faculty of Chemistry, Moscow State University, 119991 Moscow, Russia

**Keywords:** polyacrylonitrile, pore size, membrane, ultrafiltration, asphaltenes, oil fractionation

## Abstract

In this study, ultrafiltration membranes were developed via a nonsolvent-induced phase separation method for the removal of asphaltenes from crude oil. Polyacrylonitrile (PAN) and acrylonitrile copolymers with acrylic acid were used as membrane materials. Copolymerizing acrylonitrile with acrylic acid resulted in an improvement in the fouling resistance of the membranes. The addition of 10% of acrylic acid to the polymer chain decreases the water contact angle from 71° to 43°, reducing both the total fouling and irreversible fouling compared to membranes made from a PAN homopolymer. The obtained membranes with a pore size of 32–55 nm demonstrated a pure toluene permeance of 84.8–130.4 L/(m^2^·h·bar) and asphaltene rejection from oil/toluene solutions (100 g/L) of 33–95%. An analysis of the asphaltene rejection values revealed that the addition of acrylic acid increases the rejection values in comparison to PAN membranes with the same pore size. Our results suggest that the acrylonitrile–acrylic acid copolymer ultrafiltration membranes have promising potential for the efficient removal of asphaltenes from crude oil.

## 1. Introduction

Crude oil is a complex substance of compounds that contains various fractions, including the heaviest fraction known as asphaltenes [1]. Asphaltenes are high molecular-weight, polar compounds that contain numerous polycyclic aromatic or naphthenic rings. Due to their composition, asphaltenes exhibit solubility in aromatic hydrocarbons and low solubility in alkanes [2]; hence, the mixing of oils from different fields triggers the aggregation of asphaltene molecules with the formation of insoluble particles 5–300 nm in size [3]. The deposition of asphaltenes in pipelines can result in a reduction in their capacity and can cause equipment damage.

Depending on the crude oil source, the molecular weight of asphaltene molecules ranges from 250 to 1200 g/mol [1,2,4,5]. Asphaltene molecules are heterogeneous in terms of their chemical structure. They contain heteroatoms (N, O, S) and metals (V, Fe, Ni, etc.) in their structure [2,6]. Complexes of these metals, porphyrins in particular, occur in abundance in asphaltene deposits and make a significant contribution to the formation of deposits on pipelines [2]. Due to the wide variety of asphaltenes’ molecular structure, there are multiple models of asphaltenes’ molecular structure [1].

Another type of high molecular-weight polar compound in crude oil is resins. Resins have a profound influence on the stability behavior of asphaltenes and can prevent the separation of the asphaltene molecules. In terms of their structure, resins are very similar to asphaltenes, but they have a lower molecular weight (MW) (<1000 g/mol) and a higher hydrogen/carbon (H/C) ratio [2].

With the depletion of oil fields, the proportion of the high molecular fractions in crude oil increases. Consequently, there is growing interest in developing methods for deasphalting heavy oils. In recent years, much attention has been paid to the removal of asphaltenes during the on-site processing of heavy, high-viscosity oils with high asphaltene content. The existing methods for the removal of asphaltenes (deasphalting) can be conditionally divided into four groups: solvent extraction, adsorption, thermal catalytic, and chemical [7,8,9]. Solvent deasphalting is the main physical process through which asphaltene is separated from the heavy feedstock by mixing with single or a mixture of alkanes. The adsorption method consists of the use of adsorbents in the deasphalting process. In thermal catalytic deasphalting, asphaltenes are precipitated under high pressure at an elevated temperature in the presence of a catalyst and hydrogen. The chemical method is based on the treatment of oil with acids.

As an alternative, methods based on the use of surfactants [10], polymeric inhibitors [11], and various adsorbents, including mineral surfaces [12], metal surfaces [13], and nanoparticles [14] have also been investigated. In addition to solvent and adsorption methods for the removal of asphaltenes, membrane methods are also considered due to their simplicity, scalability, environmental friendliness, and energy efficiency.

Membranes were first used for asphaltene removal from oil in 1983 [15]. Ceramic membranes with a pore size from 20 nm to 1.4 μm were used for the separation of asphaltenes from oil from the Cold Lake heavy oil field. The retention of asphaltenes reached 80% [15]. An increase in temperature up to 190 °C makes it possible to reduce the viscosity of the oil and increase the size of asphaltene aggregates. As a result, microfiltration membranes can be used for asphaltene removal. At the same time, strong membrane fouling was observed. A similar result was obtained during the filtration of Iranian crude oil with an asphaltene content of 1–10% through ceramic membranes with a pore size of 50 and 200 nm at a temperature of 75–190 °C [16,17]. The retention of asphaltenes was 60–87%. 

Polymeric membranes were used for asphaltene removal from toluene solutions. In this case, the process temperature is limited by the thermal stability of the polymer. In [18], polyethersulfone membranes manufactured by NADIR (France) with an MWCO value of 20,000 g/mol were used for the fractionation of asphaltenes of various sizes. Marques et al. [19] used a combination of the aforementioned ultrafiltration membrane and Starmem 240 nanofiltration membranes made of polyimide with an MWCO value of 400 g/mol for asphaltene size polydispersity reduction. The study showed that as a result of filtration through the ultrafiltration membranes, there was a significant decrease in the concentration of asphaltenes to a level of 1.7–7.8% of the initial level. In this case, there is a significant decrease in the proportion of components with a molecular weight of more than 1000 g/mol. In solutions that have been filtered through a nanofiltration membrane, only components with a molecular weight of 200–300 g/mol or lower remained.

In [20], the separation of asphaltenes from various oils with an asphaltene content of 0–7.6% was investigated using polytetrafluoroethylene (Gore-Tex) membranes with a pore size of 30 nm and a process temperature of 80 °C. It was shown that, as a result of filtration, there is no change in the concentration of asphaltenes, from which the authors concluded that the size of asphaltene aggregates in the studied oils is less than 30 nm. On the other hand, in [21], polytriazole nanofiltration membranes were used for the fractionation of pure Arabian superlight crude oil, as well as solutions of this oil in toluene in a ratio of 1:40 and 1:1 and at a temperature of 30–150 °C. The membranes retained most of the components from C_20_. An increase in the process temperature from 80 to 150 °C led to an increase in retention, resulting in a permeate consisting of 90% hydrocarbons below C_10_. Dilution with toluene makes it possible to control the degree of retention of various oil fractions; thus, during filtration of oil/toluene solutions, the retention for the kerosene fraction (C_17_–C_25_) was lower, while the membrane completely retained the asphaltenes. 

In [22], polyacrylonitrile (PAN) membranes were used for the selective separation of asphaltenes of the “continent” type, which are prone to aggregation from molecules of the “archipelago” type. In this work, model solutions of asphaltenes in toluene, as well as solutions of fuel oil in toluene, were filtered through PAN membranes with pore sizes of 10 and 17 nm. With an overall relatively low asphaltene retention of 35–67%, the agglomerates were retained with an efficiency of 90%, which makes it possible to separate the molecules most prone to precipitation [22].

Polyacrylonitrile (PAN) is a widespread polymeric material. It is a semicrystalline polymer that is insoluble in nonpolar and low-polar solvents, such as hydrocarbons and alcohols [23]. PAN finds a wide range of applications, from the textile industry [24], through polymeric membranes preparation [25,26], to the production of carbon materials [27]. 

PAN possesses good mechanical properties, resistance to many classes of solvents, thermal and chemical stability, and low cost [28]; however, a significant challenge when using membranes for oil filtration is membrane fouling. Compared to other membrane materials, such as polyvinylidene fluoride, polysulfone, and polyethersulfone, PAN is less prone to fouling with organic compounds [29]; hence, PAN membranes exhibit a promising combination of chemical and fouling resistance toward hydrocarbons. The addition of a volatile co-solvent into the casting solution has been found to decrease the molecular weight cut-off (MWCO) of PAN membranes down to 1800 g/mol [30], which is comparable to the molecular weight of asphaltenes.

It has been shown that the permeance of PAN membranes rapidly decreased during the filtration of an asphaltenes solution and heavy oil [22]. This indicates the need for further enhancement of the fouling resistance of PAN. The main strategy to improve the fouling resistance towards hydrocarbons is through membrane hydrophilization. Modification methods applied to improve the fouling resistance and separation performance can be divided into several of the most common techniques: modification of the membrane surface [31], chemical modification [32], and addition of various nanoparticles [33]. The most popular strategy is the addition of nanoparticles due to the wide variety of possible fillers. Materials such as graphene oxide [34], carbon nanotubes [35], activated carbon [36], nanodiamonds [37], silver [33] and copper salts [38], titanium oxide [39], silica particles [40], alumina [41], and iron [42] have been used as fillers.

The addition of hydrophilic oligomers into the casting solution is another widely investigated way to improve the fouling resistance of the membranes [43]. The addition of polyethylene glycol (PEG) or glycerol is a commonly used method to regulate the porous structure of membranes prepared via a nonsolvent-induced phase separation method, and these components also improve the fouling resistance of the obtained membranes [44]. 

Another approach to enhance the hydrophilicity and fouling resistance of PAN membranes is through modifications to the synthesis process. Copolymerization of the basic polymer with hydrophilic monomers modifies the surface properties while retaining the advantages of the basic polymer. Commercial PANs are often copolymers with methyl acrylate or itaconic acid [45], which improves their mechanical properties. Copolymerization with methacrylic acid was also used for modification of an acrylonitrile-based polymer [46]. At the same time, the antifouling properties of membrane material can be improved by the addition of a hydrophilic component [47,48]. Grafting of a PAN ultrafiltration membrane with polyethylene glycol (PEG) was used to enhance its hydrophilicity and antifouling ability toward proteins [48,49].

The incorporation of the hydrophilic component directly into the polymer network provides a promising approach to improve the fouling resistance and overcome the limitations of other modification methods, such as the poor dispersion of fillers in the polymer matrix or erosion of the hydrophilic component from the membrane surface. From this point of view, copolymerization of acrylonitrile with acrylic acid (AA) appears to be the best method to improve the antifouling performance due to the hydrophilic nature of AA [50]. The purpose of this work is to investigate the improvement of acrylonitrile-based membrane fouling resistance during the oil filtration process by copolymerization of acrylonitrile with acrylic acid.

## 2. Materials and Methods

### 2.1. Materials

Acrylonitrile monomer (>99.5) was purchased from Fluka (Buchs, Switzerland). Dimethyl sulfoxide, N-methyl-2-pyrrolidone (NMP) (>99%), dimethylformamide (DMF) (>99%), ammonium peroxodisulfate, sodium dithionite, sulfuric acid, and potassium carbonate were acquired from Khimmed (Moscow, Russia). Helium with a purity of at least 99.95% was purchased from the Moscow Gas Refinery Plant (Moscow, Russia).

### 2.2. Polymer Synthesis

Copolymers of AN and AA (P(AN-co-AA)) were synthesized in an aqueous medium in the presence of a redox system of ammonium peroxodisulfate and sodium dithionite as initiators. The copolymers were obtained at three molar ratios of AN:AA monomers: 90:10, 95:5, and 99:1. To synthesize the copolymer, sulfuric acid and monomers were added to a flask containing 300 mL of bidistilled water: [H_2_SO_4_] = 1.9 × 10^−2^ mol/L, [AN] = 1.14, 1.20, or 1.25 mol/L, [AA] = 0.13, 0.06, or 0.01 mol/L for ratios of 90:10, 95:5, or 99:1, respectively. The initiators were added simultaneously in the following concentrations: [(NH_4_)_2_S_2_O_8_] = 3.92 × 10^−3^ mol/L, [Na_2_S_2_O_4_] = 1.68 × 10^−3^ mol/L. The flask was shaken and placed in a thermostat for 40 min at 60 °C. Then, 100 mL of the water solution containing the monomers and sulfuric acid was added to the existing emulsion. The concentrations of the components were as follows: [H_2_SO_4_] = 1.9 × 10^−2^ mol/L, [AN] = 1.78, 1.88, or 1.96 mol/L, [AK] = 0.2, 0.1, or 0.02 mol/L for ratios of 90:10, 95:5, or 99:1, respectively. After the addition of the monomers, the reaction continued for 2 h. The polymer was filtered and washed in water and ethanol to remove the sulfuric acid, oligomers, and monomer residues. Then, it was dried in a vacuum to a constant weight.

A laboratory-synthesized PAN homopolymer was used as a reference material. This polymer was used for preparation of the membrane in our previous works and a detailed description can be found in our previous articles [25,30,51]. Membranes from this polymer were used as a reference for comparison of the obtained P(AN-co-AA) membranes.

### 2.3. Polymer Characterization

The study of the molecular weight characteristics of the resulting polymer was carried out by gel permeation chromatography (GPC) on a GPC-120 chromatograph (PolymerLabs, Santa Clara, CA, USA). The analysis was carried out at 50 °C in DMF. The characteristics of the obtained copolymers are presented in Table 1.

Infrared (IR) spectra registration was carried out in the reflection mode using an IR microscope HYPERION-2000 conjugated with an IR-Fourier spectrometer IFS 66 v/s Bruker (Ge crystal, scan-50, resolution 2 cm^−1^, range 600–4000 cm^−1^) (Bruker Physik AG, Karlsruhe, Germany).

Kinetic and thermochemical dependences were studied by thermogravimetric analysis (TGA) and differential scanning calorimetry (DSC) in the temperature range of 40–450 °C and 40–400 °C, respectively, using Discovery TG TM (TA Instruments, New Castle, DE, USA) and Q20 (TA Instruments, USA). The heating rate was 10 °C/min at an argon flow rate of 50 mL/min. The sample weights ranged from 2 to 4 mg.

X-ray diffraction spectra were obtained using an X-ray diffractometer Difray-401 (Scientific Instruments, Saint Petersburg, Russia) at room temperature on Cr-Kα radiation.

### 2.4. Solution Preparation and Characterization

In the case of P(AN-co-AA), the concentration in the casting solution was kept constant at 12% using NMP as a solvent. This value was chosen based on the consideration that at higher concentrations, the solution’s viscosity became too high to achieve consistent membrane preparation. The PAN concentrations in the NMP solutions ranged from 12 to 20 wt.%, covering a wide range of pore sizes and permeances.

The solution preparation was carried out at a temperature of 20 °C and humidity of 20%. The solution was treated in an ultrasonic bath for 30 min with further stirring for 7 days at 45 °C to produce a homogeneous solution. The obtained solution was treated in an ultrasonic bath again for 30 min to complete homogenization and remove air bubbles. The prepared solution was stored in a hermetically sealed flask at 20 °C and 20% humidity in darkness.

The dynamic viscosity of the prepared solutions was measured at 20 °C using a Brookfield DV2T-RV viscometer (Ametek Brookfield, Middleboro, MA, USA).

### 2.5. Membrane Preparation

The membranes were prepared by the NIPS method with water as a nonsolvent. The polymer solution was cast on a glass plate with a thickness of 200 μm at a casting speed of 2.5 m/min. The membrane casting was carried out at 20 °C and 20% humidity. The cast film was immediately immersed in the coagulation bath (distilled water, 20 °C) for 24 h. After completion of the membrane formation, the samples were washed with distilled water and kept in a new portion of distilled water for 24 h to wash out the solvent residues. Then, the membrane was placed in ethanol for at least 24 h, after which the membrane was placed in isobutanol for another 24 h. The excess liquid was removed from the film with filter paper and left between two sheets of filter paper until completely dry. From each casting solution, three membranes were prepared under identical conditions. The results presented in this work are averaged for at least three identical membranes.

### 2.6. Membrane Characterization

The filtration experiments were carried out in a dead-end stirred filtration cell. The membrane sample was placed on the porous stainless-steel disks and sealed with rubber O-rings. The active membrane area was 7.9 cm^2^. At least three sections were measured from every membrane, and the permeance data were averaged. The difference in permeance measured for different membrane sections was up to 10%. The system was pressurized with helium. The membrane permeance was measured from the beginning of the experiment without initial pre-pressurization. Permeate samples were taken every 5 min until a constant permeance was obtained, and then 5 more measurements were taken at 5 min intervals. The amount of collected permeate per interval depends on the membrane permeance. In the case of pure water and toluene, the permeate ranged from 50 to 100 mL.

The sample permeance was determined as an average for the last 5 data points. In the case of solution filtration, the feed sample was stirred at 600 rpm to minimize the concentration polarization effect. The membrane permeance *L_p_* (L/(m^2^·h·bar)) was determined as:

(1) LP=J∆p=mρ·S·t·∆p

where *J* is the flux of the liquid, ∆*p* is the transmembrane pressure (bar), *m* is the mass of the permeate (g), *ρ* is the density of the permeate (g/L), *S* is the active membrane area (m^2^), and *t* is the filtration time (h). The transmembrane pressure was 5 bar.

For every membrane, the water permeance was measured. The oil solution in toluene with an oil content of 1, 10, and 100 g/L was filtered through a separate section cut from the same membrane. The pure toluene permeance was then determined before and after the oil solution filtration. Oil from the Salym field was used in this work. The asphaltene content in the used oil was ~5%. To prepare the solutions, the oil was poured into a beaker and weighed, and then the toluene was added. As a result, solutions were obtained with an asphaltene concentration in the range of 0.5–5 g/L. The solutions were left to mix for several hours on a magnetic stirrer. After toluene filtration, 500 mL of an oil solution in toluene was poured into the cell and electromagnetically stirred. Five minutes later, a sample of the solution was taken from the cell for further analysis. The system was pressurized after the feed sample was collected from the cell. In the process of the oil solution filtration, 10 samples of permeate with a volume of 10 mL each were taken. The collection of 10 mL of the permeate took anywhere from 1 to 10 min in these experiments depending on permeance. After the filtration process was stopped, a sample of retentate was taken from the cell. After completion of the oil solution filtration, the cell was washed three times with 50 mL of toluene, after which 800 mL of toluene was refilled into the cell, and the filtration was carried out until a constant toluene flux was observed.

The membrane rejection was estimated using solution optical density. This parameter was measured using a PE-5400UF spectrophotometer (PromEcoLab, Shanghai, China). Toluene was used as a reference solution. As the wavelength increased, the optical density of the solutions decreased monotonically. A fixed wavelength was used for the rejection calculation depending on the initial solution composition. If the initial solution was 1 g/L, then the feed, retentate, and permeate samples were measured at 420 nm. Consequently, for 10 g/L, the samples were measured at 550 nm, and for 100 g/L, the samples were measured at 930 nm. The same wavelength was used for liquid collected from the filtration cell at the end of the experiment. The specified wavelengths were chosen because the corresponding initial solutions had an optical density close to 1. These wavelengths were used with the spectrophotometer to measure the optical density from 0.001 to 3; hence, both the feed and permeate samples can be measured at the same wavelength.

The rejection *R* was calculated using the following equation:

(2) R=1−CpCf·100%

where *C_f_* and *C_p_* denote the solute concentrations in the feed and permeate, respectively.

It should be noted that a wide range of components contribute to the optical density; hence, changes in the optical density cannot be attributed to the rejection of a certain component. At the same time, preliminary experiments with a solution of asphaltenes and various petroleum products show that the asphaltenes absorb the largest fraction of light and, hence, contribute the most to optical density. Resins also contribute to optical density, but their impact is significantly less. The optical density of light hydrocarbons is comparable to the optical density of toluene in the studied wavelength range; therefore, their contribution can be neglected. Thus, the rejections determined based on the change in optical density can be roughly attributed to the retention of the asphaltene fraction, although the membrane can retain not only asphaltenes, but also other smaller components, which also make a contribution to the optical density value.

The calculations in this article using the optical density can be attributed to the asphaltenes’ fraction rejection.

To evaluate the fouling behavior of the membrane, the flux recovery ratio (*FRR*) and several resistance ratios, namely, the total fouling ratio (*TFR*), reversible fouling ratio (*RFR*), and irreversible fouling ratio (*IFR*), were calculated according to the following equations:

(3) $$FRR=\frac{J_{2}}{J_{1}}\cdot 100\%,$$

(4) $$TFR=\left(\frac{J_{1}-J_{S}}{J_{1}}\right)\cdot 100\%,$$

(5) $$RFR=\left(\frac{J_{2}-J_{S}}{J_{1}}\right)\cdot 100\%,$$

(6) $$IFR=\left(\frac{J_{1}-J_{2}}{J_{1}}\right)\cdot 100\%,$$

where *J*_1_ is the initial pure toluene flux, *J*_2_ is the pure toluene flux through the fouled membrane, and *J_S_* is the oil solution flux.

The pore size was measured by a liquid–liquid displacement porosimetry (LLDP) [52,53] using the porometer POROLIQ 1000 ML (Porometer, Nazareth, Belgium). The operating principle is based on the measurement of the equilibrium pressure corresponding to the flux of the displacing liquid. The displacement of the wetting liquid was carried out by a stepwise increase of the transmembrane pressure with monitoring of the flux through the membrane after 180 s initial stabilization time at each applied pressure. The measurement was stopped after reaching a linear dependence of the flux on the pressure, which indicated a complete displacement of the wetting liquid. The alcohol-rich phase was used as the wetting liquid and the water-rich phase was used as the displacing liquid. Three sections (2 cm in diameter) were cut from every membrane and were placed into the beaker with the wetting liquid for at least 2 h at 20 °C before testing. The results were averaged for all the investigated samples. The measurements were carried out at 25 °C using a pair of immiscible liquids prepared by demixing a mixture of isobutanol and water (1/4, *v*/*v*). The diameter (*D*) of the open pore is related to the transmembrane pressure via the Young–Laplace equation:

(7) D=4·γ·cos⁡θ∆p

where *γ* is the interfacial tension between the two liquids, *θ* is the contact angle between the membrane and the wetting liquid (complete wetting is assumed, i.e., cos *θ* = 1), and Δ*p* is the transmembrane pressure. The interfacial tension *γ* for the mixture of isobutanol and water is 1.9 mN/m at 25 °C. The pore size was calculated according to the procedure described in detail in [53].

The measurements of the contact wetting angles were performed by the standard method of a lying drop using an LK-1 goniometer manufactured by RPC OpenScience Ltd. (Krasnogorsk, Russia). The measurements were carried out on the side of the selective layer. Data acquisition and subsequent digital processing of the droplet images for the direct calculation of angles using the Young–Laplace equation were carried out using DropShape software. The measurement error was ±2°. The measurement temperature was 23 ± 2 °C.

Scanning electron microscopy (SEM) was used to characterize the structure and morphology of the membranes. SEM was carried out on a Thermo Fisher Phenom XL G2 Desktop SEM (Thermo Fisher Scientific, Waltham, MA, USA). Cross-sections of the membranes were obtained in liquid nitrogen after preliminary impregnation of the specimens in isopropanol. A thin (5–10 nm) gold layer was deposited on the prepared samples in a vacuum chamber (~0.01 mbar) using a desktop magnetron sputter “Cressington 108 auto Sputter Coater” (Cressington Scientific Instruments, Watford, UK). The accelerating voltage during image acquisition was 15 keV.

## 3. Results and Discussion

### 3.1. Copolymer Characterization

**Figure 1:**
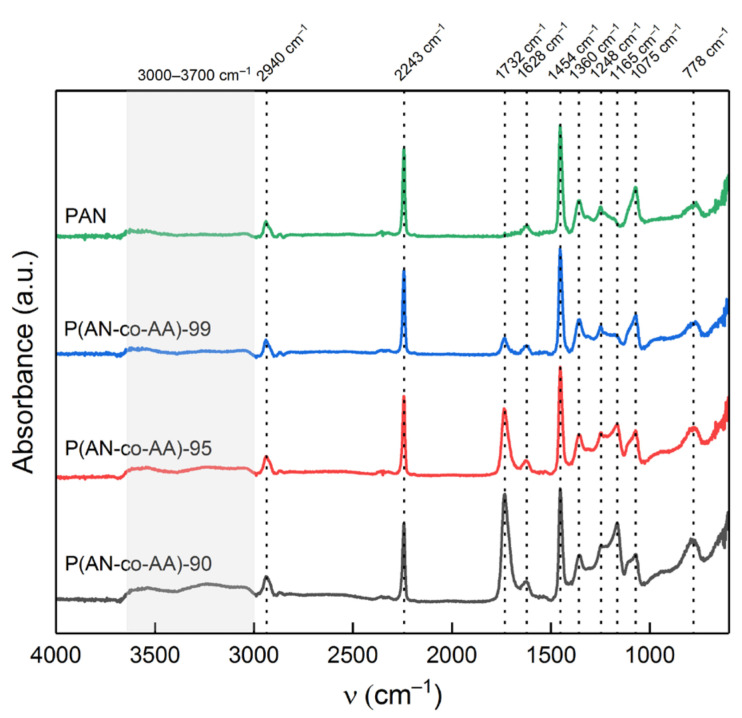
FTIR spectra of synthesized PAN and P(AN-co-AA) copolymers.

The chemical structure of the synthesized acrylonitrile homopolymer and copolymers was studied by FTIR spectroscopy (Figure 1). All the IR spectra show a characteristic band of stretching vibrations of the nitrile group (ν_C≡N_) at 2243 cm^−1^, which refers to the monomer units of acrylonitrile. Another characteristic band, indicating the presence of monomer units of acrylic acid, refers to the stretching vibrations of the carboxyl group (ν_C=O_). Its presence at 1732 cm^−1^ is observed only for the copolymer samples, which confirms the copolymerization of the initial AN and AA monomers. The nearby band at 1628 cm^−1^ can be attributed to the deformation vibrations of water [53]. The bands at 2940 cm^−1^ correspond to –CH_2_ stretching vibrations, the bands at 1360 and 1454 cm^−1^ correspond to –CH_2_ bending vibrations, and the band at 1075 cm^−1^ corresponds to mixed –CH_2_ vibrations [54,55]. The bands at 1248 and 778 cm^−1^ can be attributed to mixed vibrations of the C–H bond [55,56,57]. The band at 1170 cm^−1^, which is present only in the spectra of the copolymers, belongs to vibrations of C–O and C=O bonds in the composition of AA monomer units. The region at 3000–3700 cm^−1^ refers to –OH bonds and adsorbed water [55,58]. The intensity of this band is higher in the copolymer samples, while in the acrylonitrile homopolymer samples, it is almost absent.

**Figure 2:**
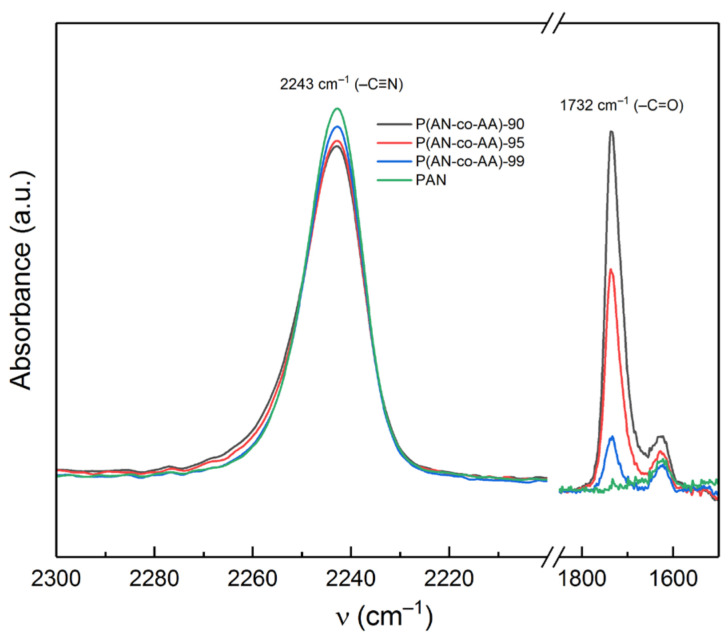
Fragments of FTIR spectra of the synthesized PAN and P(AN-co-AA) copolymers.

With an increase in the proportion of the AA monomer during the synthesis of the copolymers, the intensity of the band at 2243 cm^−1^ (ν_C≡N_) decreases, while a sharp increase in the intensity of the band at 1732 cm^−1^ (ν_C=O_) is observed (Figure 2). According to the Bouguer–Lambert–Beer law, the absorption value is proportional to the concentration of the absorbing substance; thus, by the ratio of the intensities of the absorption bands from the C≡N and C=O bonds, one can estimate the compositional composition of the P(AN-co-AA) copolymers [55,59]. In [56], the dependence of the intensity ratio of the A_1732_/A_2243_ bands on the content of the AN and AA homopolymers in the mixture was studied. The formula A = 0.18 + 0.15x derived from the dependence graph allows us to estimate the content of acrylic acid monomer units in the AN and AA copolymer. In the case of the P(AN-co-AA)-95 copolymer, the content of the monomer units was calculated to be 4.37 mol.%, which closely matches the initial fraction of the AA monomer (Table 2).

The estimated values of AA are close to the molar concentrations of the monomer used in the reaction mixture; however, significant discrepancies between the actual and calculated values for the P(AN-co-AA)-99 sample indicate that the calculation of the copolymer composition using the given equation introduces a certain margin of error.

**Figure 3:**
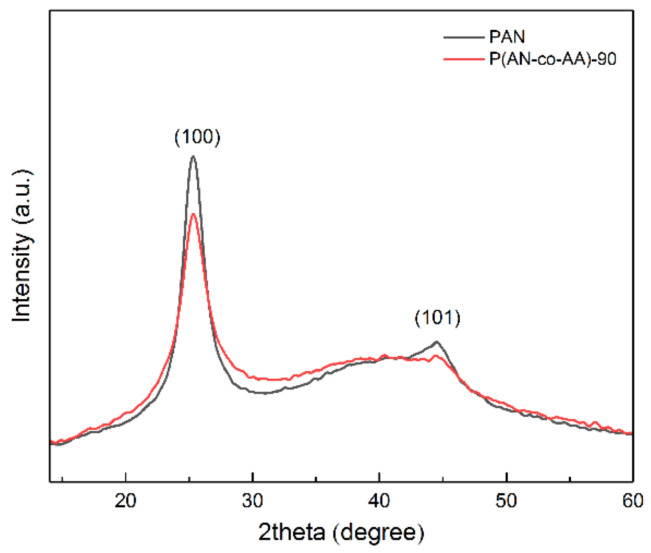
X-ray diffraction patterns of PAN and P(AN-co-AA)-90 samples.

In the XRD patterns of the acrylonitrile homopolymer, the most intense peak is observed at 2*θ* = 25.3° (Figure 3), which corresponds to the (100) plane of the hexagonal lattice of the polymer. The second peak corresponding to the (101) plane is observed at 2*θ* = 44.6°. In this case, the intensity of the crystalline peaks of the PAN sample is the highest, which indicates a higher crystallinity of this polymer. The addition of AA leads to a decrease in the crystallinity of the polymer. The lowest crystallinity corresponds to the P(AN-co-AA)-90 copolymer with the highest content of AA monomer units. The side groups of P(AN-co-AA) interfere with the intermolecular interaction of the nitrile groups [55,60], which leads to a violation of the order of macromolecules.

**Figure 4:**
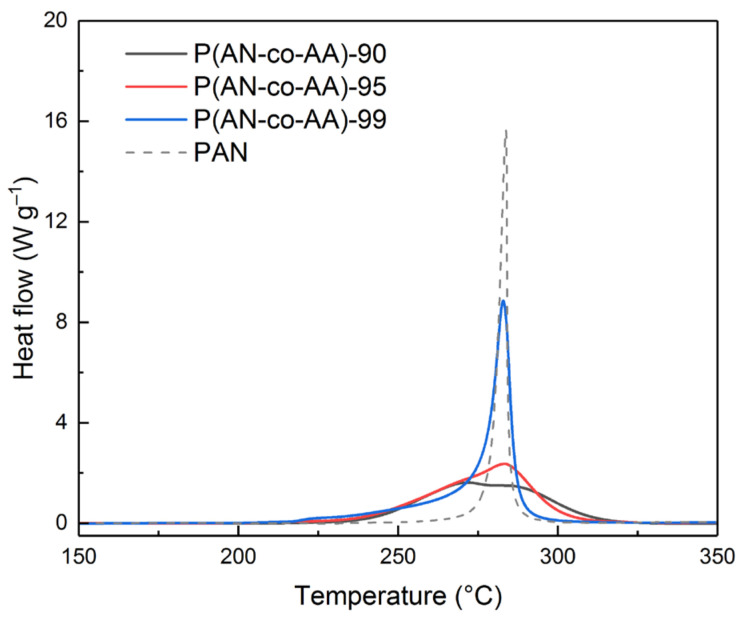
DSC results for PAN and P(AN-co-AA) samples.

An analysis of the DSC results (Figure 4) showed that with an increase in the proportion of the acrylic acid monomer, the intensity of the peak of the thermal effect, which occurs during the cyclization of copolymers in an inert atmosphere, significantly decreases. Even the addition of 1% AA leads to a decrease in the maximum exothermic effect of cyclization by 1.8 times. In this case, asymmetry of the peak on the side of low temperatures begins to appear. The addition of 5% AA to the polymerization mixture leads to a decrease in the corresponding peak of the copolymer by more than 6.5 times compared to PAN. A split of the peak into two is observed for P(AN-co-AA)-90. The low-temperature peak corresponds to the cyclization of the copolymer according to the ionic mechanism characteristic of AA. The high-temperature peak corresponds to the radical mechanism characteristic of PAN. An increase in the AA content from 5 to 10% leads to less significant changes in the thermogram, but the effect of AA on the cyclization processes is more pronounced. The intensity of the exothermic peak corresponding to the ionic mechanism of cyclization of the copolymer increases, and the intensity of the peak responsible for the radical mechanism of cyclization decreases.

The addition of AA leads to a shift in the maximum of the exothermic peak corresponding to the radical mechanism of copolymer cyclization towards an increase in the temperature from 282 to 286 °C, while the maximum of the exothermic peak of AA falls at 272 °C, which is in good agreement with results obtained earlier in [61].

An increase in the AA content from 1 to 10% generally leads to a decrease in the thermal effects of the cyclization process from −589.4 to −444.7 J/g (144.7 J/g). At the same time, for PAN, the thermal effect is comparable (−441 J/g) at a narrower temperature range of the cyclization process (73 °C) (Table 3). The addition of AA monomeric units in small concentrations to the polymer chain leads to an increase in the heat effect of the cyclization process, but the process starts at a lower temperature. It may indicate the initiating effect of the carboxyl group of the AA monomeric unit on the process. A further increase in the AA content leads to a decrease in thermal effects with a simultaneous decrease in the temperature at the beginning of the process due to an increase in the influence of the ionic cyclization mechanism.

**Figure 5:**
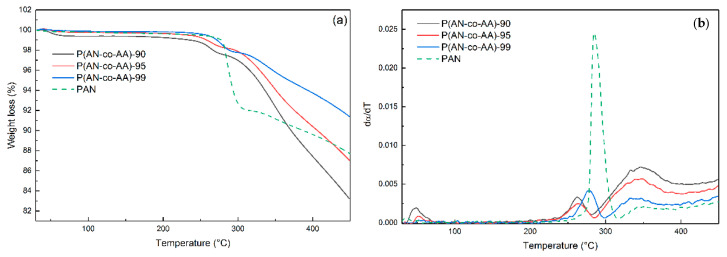
Dependence of weight loss (**a**) and derivative of the conversion degree (**b**) on heating temperature of copolymers.

The TGA results analysis (Figure 5) revealed distinct features for the copolymers compared to the homopolymer. The copolymers exhibited more uniform mass loss dynamics, unlike PAN, which showed a sharp mass change in the range of 270–290 °C due to intense thermal transformations and polymer cyclization, accompanied by an exothermic effect. Moreover, the copolymers exhibited lower weight loss up to a temperature of 360 °C. With an increase in the AA content in the polymer chain, the weight loss increases. At the same time, for the P(AN-co-AA)-90 sample, a decrease in weight by 0.8% is observed in the range of 40–70 °C, which may be due to the desorption of water molecules, which indicates greater hydrophilicity compared to PAN.

An analysis of the temperature dependence of the derivative of the conversion degree in the range of 200–320 °C (Figure 5b) revealed that the addition of AA resulted in a decrease in the temperatures of the onset of cyclization. The maximum mass loss rate for P(AN-co-AA)-99 was observed at a temperature of 277 °C, which is 6 °C lower than for PAN. A further increase in the AA content to 10% leads to a decrease in temperature to 262 °C, which is determined by the action of the ionic cyclization mechanism (low temperature). At temperatures of 320–450 °C, the intensification of the mass loss process is observed for the copolymers, which is presumably associated with the release of CO and CO_2_ from the AA units.

The main factor of membrane fouling in oil filtration is the deposition of asphaltenes onto the membrane surface and the addition of AA chains. A decline in the affinity between the membrane surface and the asphaltene molecules decreases the membrane fouling. Membrane hydrophilicity is the factor for characterization of membrane surface changes, which is indirectly linked with affinity between the membrane surface and the asphaltenes.

**Figure 6:**
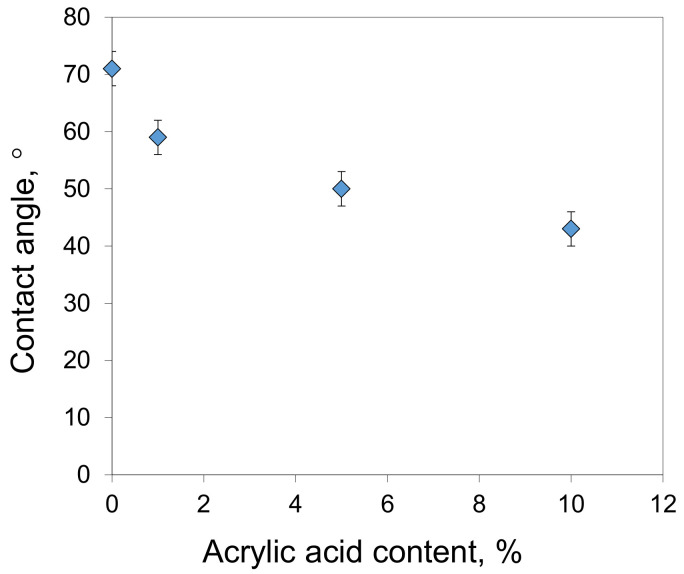
Water contact angles of membranes prepared from copolymers with different acrylic acid content.

Acrylic acid chains are hydrophilic and their incorporation into the polymer increases the hydrophilicity of the obtained membranes. The hydrophilization effect was evaluated by measuring the water contact angles of the synthesized membranes (Figure 6). The addition of 1% of AA units decreased the water contact angle by 12° from 71 ± 3° to 59 ± 3°. For copolymers consisting of 10% AA units, the water contact angle was 43 ± 3°, which is 28° lower than that of the PAN membranes. This increase in hydrophilicity is important for the antifouling properties of the obtained membranes, as it helps to prevent the deposition of rejected components on the membrane surface.

### 3.2. Membrane Preparation and Characterization

**Figure 7:**
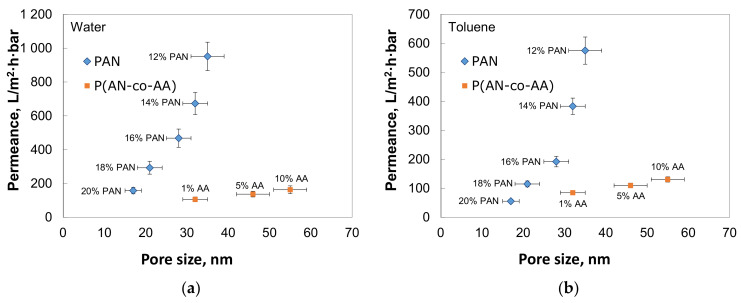
Permeance of water (**a**) and toluene (**b**) through membranes from PAN and P(AN-co-AA).

**Figure 8:**
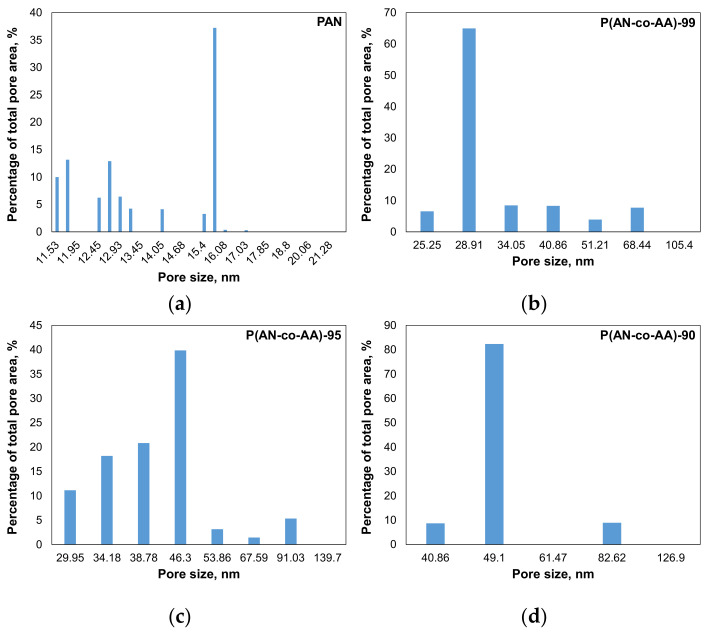
Examples of raw data of obtained pore size distributions for 20% PAN (**a**), P(AN-co-AA)-99 (**b**), P(AN-co-AA)-95 (**c**), and P(AN-co-AA)-90 (**d**).

The membranes from the synthesized copolymers and PAN were obtained. Increasing the AA content in the copolymer increased the pore size and permeance. The membrane prepared from a copolymer containing 10% AA using a 12% NMP solution exhibited a toluene permeance of 130.4 ± 9.1 L/(m^2^·h·bar) and a pore size of 55 ± 4 nm (Figure 7). In comparison, the membrane prepared from P(AN-co-AA)-99 showed a toluene permeance of 84.8 ± 7.6 L/(m^2^·h·bar) and a pore size of 32 ± 3 nm. All the membranes demonstrated quite narrow pore size distribution, but the P(AN-co-AA) membranes also have some pores with a size greater than the average pore size (Figure 8).

**Figure 9:**
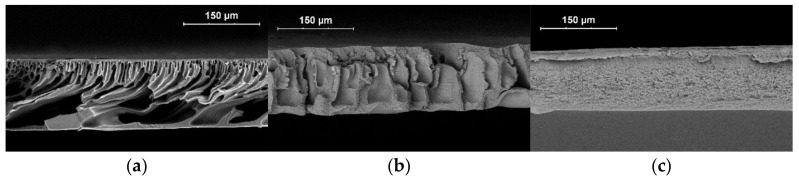
SEM images of the membrane cross-section with respect to the casting solution composition: (**a**) PAN; (**b**) P(AN-co-AA)-99; (**c**) P(AN-co-AA)-90.

The SEM images of the membranes’ cross-sections (Figure 9) demonstrate that the addition of 1% of AA promotes the formation of a spongy structure, whereas all the prepared PAN membranes show a finger-like porous structure with a thin selective layer. Such a structure possesses higher permeance of the PAN membranes despite their small pore size. The membranes prepared from the copolymer with 10% AA show a spongy structure without macrovoids (Figure 9). At the same time, membranes with 1% of AA have irregular macrovoids, although their permeance is also low (Figure 7). Apparently, the thick spongy layer on the P(AN-co-AA)-99 membrane has the greatest impact on membrane permeance.

The PAN membranes had finger-like pores with a dense top layer when P(AN-co-AA)-90 had a spongy structure. At the same time, the pore size in the top layer of P(AN-co-AA)-90 is larger than in the PAN dense top layer. The presence of finger-like macrovoids in the PAN membranes explains the much higher permeance of these membranes. The copolymer membranes had a denser structure without finger-like pores. The thick spongy layer in the P(AN-co-AA) membranes leads to lower permeance due to a denser structure and possibly more closed porosity formed due to the incorporation of AA units.

The difference in the membrane structure can be connected to the dope solution viscosity and changes in the polymer–solvent–precipitant interaction due to the influence of the AA chains. According to a previous investigation by our colleagues, higher solution viscosity results in the formation of a spongy structure. The higher molecular weight of the polymer results in increased solution viscosity, thereby altering the phase separation process. Due to the high molecular weight of the synthesized copolymers, a limited range of concentrations can be used for membrane formation, which restricts the optimization of membrane characteristics. The P(AN-co-AA) solutions had very high solution viscosities of 187.3 ± 1.4, 141.7 ± 1.5, and 135.9 ± 1.2 Pa·s for P(AN-co-AA)-99, 95, and 90, respectively. For the PAN solutions, the viscosity varied in a range from 3.9 ± 0.7 Pa·s for the 12% solution to 70.6 ± 1.0 Pa·s for the 20% solution, which is much lower. The high viscosity of the casting solution promotes the formation of spongy structures obtained for membranes from the copolymers. At the same time, the viscosity of the P(AN-co-AA)-90 solution was lower than that of P(AN-co-AA)-99; thus, the difference in viscosity does not explain the change in the membrane structure between P(AN-co-AA)-99 and P(AN-co-AA)-90. At the same time, the increase in the AA content also promotes the formation of a spongy structure. Hydrophilic AA chains can make water a less hard precipitant. Since more water uptake is needed to start phase separation, phase separation occurs later and goes in another direction. As a result, an increase in the AA content promotes the formation of a spongy structure despite some decrease in the solution viscosity.

### 3.3. Oil/Toluene Solution Filtration

**Figure 10:**
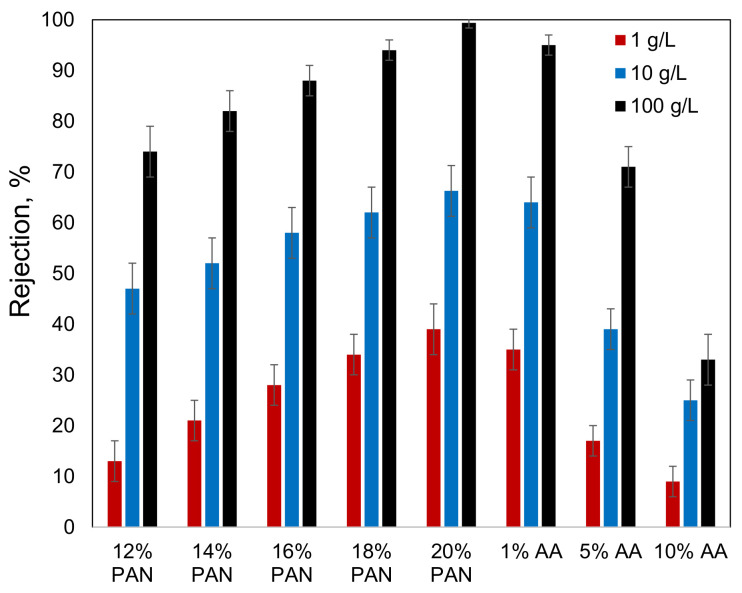
Rejection of asphaltenes from oil/toluene solutions by different membranes.

The filtration performance of the membranes prepared from the copolymers was evaluated by filtering the oil/toluene solutions. The results have shown that an increase in the AA content leads to a decrease in membrane rejection (Figure 10). On the other hand, the P(AN-co-AA)-99 membrane demonstrated higher rejection than the membrane prepared from the 12% PAN solution, which was the same concentration used for the P(AN-co-AA) membrane preparation. A comparison of the results obtained at different oil concentrations reveals that increasing the oil content leads to an increase in membrane rejection. This observation is associated with an increase in the size of the asphaltene aggregates with an increase in their concentration. A higher oil content in the filtered solution means a higher asphaltene concentration and, hence, bigger particles to be rejected by the membrane. The fact that both the variation in the membrane pore size and oil content influence the membrane retention confirms that the size of the asphaltene aggregates is comparable to the pore sizes in the obtained membranes.

**Figure 11:**
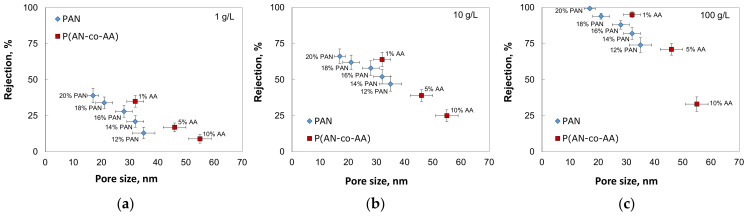
Rejection of asphaltenes as a function of membrane pore size with different oil content: (**a**) 1 g/L; (**b**) 10 g/L; (**c**) 100 g/L.

The decrease in the membrane rejection with an increase in the AA content can be explained by the increase in the membrane pore size. The comparison of the membranes’ rejection with their pore sizes revealed an interesting effect that the P(AN-co-AA)-99 membranes demonstrated higher rejection than PAN membranes with a comparable pore size (Figure 11). The mechanism behind this increase in membrane rejection requires further investigation and will be conducted in our future work.

### 3.4. Antifouling Properties of Obtained Membranes

**Figure 12:**
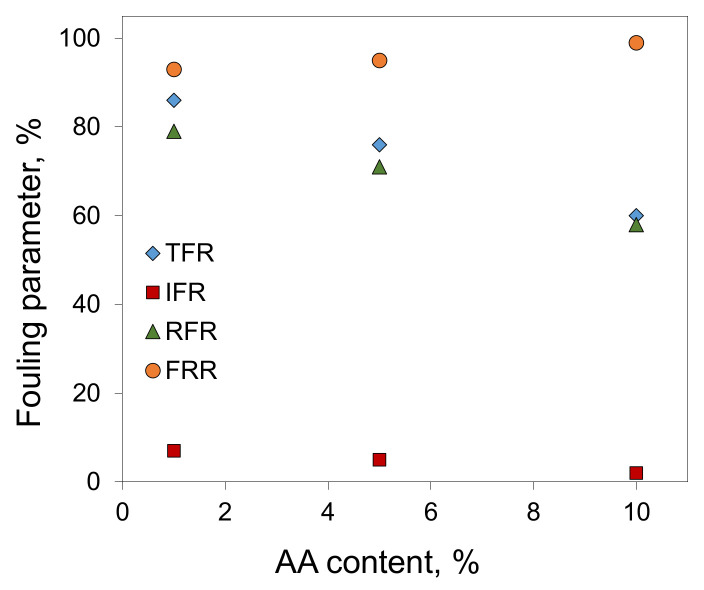
Fouling parameters of membranes from P(AN-co-AA) copolymers in the case of filtration of oil/toluene solutions (10 g/L).

In the case of filtration of oil/toluene solutions, the calculation of the fouling parameters for the P(AN-co-AA) copolymers membranes showed that an increase in the AA content leads to a decrease in both the total fouling (TFR) and irreversible fouling (IFR) (Figure 12). This confirms that the addition of hydrophobic elements into the polymer chain improves the fouling resistance of the obtained membranes. When the AA content increased from 1% to 10%, the IFR decreased from 7% to 2%, indicating that permeance losses per time unit were more than three times lower.

**Figure 13:**
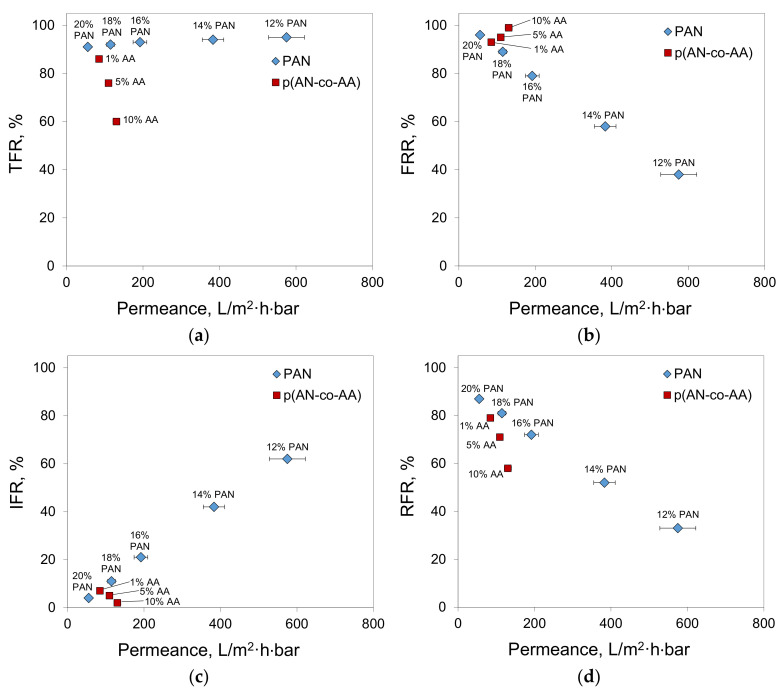
Fouling parameters of P(AN-co-AA) and PAN membranes as a function of membrane permeance (**a**) TFR; (**b**) FRR; (**c**) IFR; (**d**) RFR.

At the same time, the total fouling of all the membranes was found to be high (Figure 13a). In the case of the PAN membranes, the oil/toluene solution permeance was ten times lower than the toluene permeance. The addition of AA content decreases the TFR from 91–95% for PAN to 60% for P(AN-co-AA)-90. The inclusion of AA content also decreases the irreversible fouling, despite the tendency that an increase in the membrane permeance results in an increase in the irreversible fouling, as observed for the PAN membranes (Figure 13c). The addition of AA prevents irreversible fouling despite an increase in the membrane permeance. 

**Figure 14:**
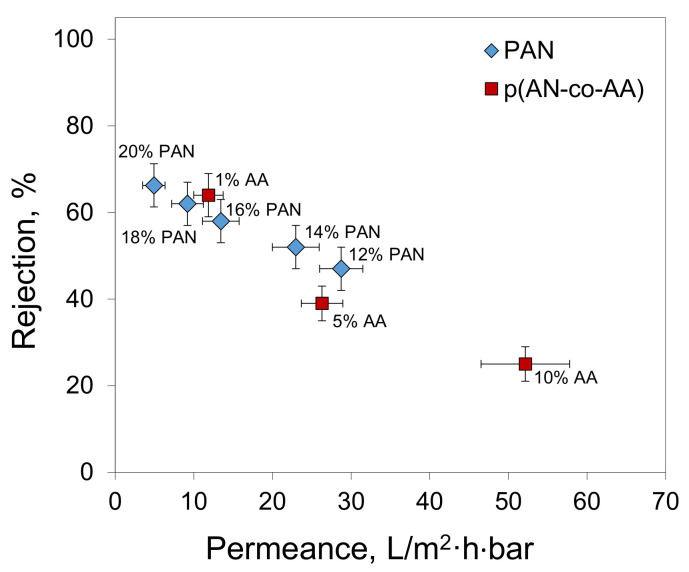
Rejection–permeance diagram for obtained membranes in the case of oil/toluene solutions (10 g/L).

Taking into account the nature of the observed dependencies, the decrease in fouling with an increase of AA content (Figure 12) correlates with an increase in the membrane hydrophilicity (Figure 6). Unlike the PAN membranes, where higher membrane permeance led to higher irreversible fouling, the P(AN-co-AA) membranes showed the opposite trend—more permeable membranes demonstrated lower IFR. This result confirms that the addition of hydrophilic chains into the polymer decreases membrane fouling during hydrocarbon separation. As a result, when compared to the PAN membranes, the P(AN-co-AA) membranes showed similar separation performance in the filtration of oil/toluene solutions (Figure 14) while exhibiting much lower fouling.

## 4. Conclusions

In summary, the present study explored the use of PAN and acrylonitrile copolymers with acrylic acid as membrane materials for the removal of asphaltenes from crude oil through ultrafiltration membranes prepared via a nonsolvent-induced phase separation method. It has been shown that the addition of acrylic acid led to a decrease in the water contact angle, indicating an increase in hydrophilicity and improved fouling resistance of the membranes during the filtration of an oil/toluene mixture. The increase in the AA content in a copolymer also promoted an increase in the pore size and permeance. Membranes prepared from P(AN-co-AA)-99 demonstrated a toluene permeance of 84.8 ± 7.6 L/(m^2^·h·bar) and a pore size of 32 ± 3 nm, while the addition of 10% AA increased these parameters up to 130.4 ± 9.1 L/(m^2^·h·bar) and 55 ± 4 nm, respectively; hence, an increase in the AA content decreased the asphaltene rejection. On the other hand, the P(AN-co-AA)-99 membrane demonstrated higher rejection than the PAN membrane prepared from the same solution composition. 

A comparison of the results obtained at different oil concentrations shows that an increase in the oil content leads to an increase in the membrane rejection. This is in agreement with an increase in the size of the asphaltene aggregates with an increase in their concentration in the solution. As a result, the rejection of asphaltenes from oil/toluene solutions with an oil content of 100 g/L reached up to 95%, depending on the membrane. It is worth noting that further optimization of the polymer synthesis conditions and membrane formation conditions can improve the membrane structure, pore size, and permeance. This is a question for our further investigations.

## Figures and Tables

**Table 1:** Characteristics of polymers used in this work.

Sample	AN:AA Ratio	Mn	Mw	Mw/Mn	Yield, %
P(AN-co-AA)-90	90:10	59,300	165,100	2.8	81.4
P(AN-co-AA)-95	95:5	73,900	257,200	3.5	82.4
P(AN-co-AA)-99	99:1	96,400	313,000	3.2	82.3
PAN	100:0	36,900	118,800	3.2	92.4

**Table 2:** Calculated AA unit content based on the intensity ratio of the A_1732_/A_2243_ bands.

Sample	A_1732_/A_2243_	Calculated AA Content, mol.%
P(AN-co-AA)-90	1.3827	8.02
P(AN-co-AA)-99	0.8361	4.37
P(AN-co-AA)-99	0.1962	0.11

**Table 3:** Thermal characteristics of PAN and P(AN-co-AA) samples.

Sample	T_max_, °C	−ΔH, J/g	Heat Flow, W/g	ΔT, °C	ΔH/ΔT
P(AN-co-AA)-90	272.3	−444.7	1.6	105	4.2
P(AN-co-AA)-95	283.4	−479.5	2.3	104	4.6
P(AN-co-AA)-99	282	−589.4	8.8	100	6.0
PAN	284	−441	15.6	73	5.5

## Data Availability

The raw/processed data required to reproduce these findings cannot be shared at this time, as the data also forms part of an ongoing study.
